# Comparison of Dosimetry Protocols for Electron Beam Radiotherapy Calibrations and Measurement Uncertainties

**DOI:** 10.3390/life12010031

**Published:** 2021-12-26

**Authors:** Fawzia E. M. Elbashir, Wassim Ksouri, Mohamed Hassan Eisa, Sitah Alanazi, Farouk Habbani, Abdelmoneim Sulieman, David A. Bradley, Ibrahim I. Suliman

**Affiliations:** 1 Department of Medical Physics, National Cancer Institute, University of Gezira, Wad Madani P.O. Box 1111, Sudan; fawzia_v@yahoo.com; 2 Department of Medical Physics, Centre de Radiothérapie Hartmann 4, Rue Kleber, CS90004, CEDEX, 92309 Levallois-Perret, France; wassim.ksouri@cea.fr; 3 Department of Physics, College of Science, Imam Mohammad Ibn Saud Islamic University (IMSIU), Riyadh 11642, Saudi Arabia; MHSalim@imamu.edu.sa (M.H.E.); sfenazi@imamu.edu.sa (S.A.); 4 Department of Physics, Faculty of Science, University of Khartoum, Khartoum P.O. Box 321, Sudan; habbanif@yahoo.com; 5 Radiology and Medical Imaging Department, College of Applied Medical Sciences, Prince Sattam bin Abdulaziz University, Alkharj 11942, Saudi Arabia; a.sulieman@psau.edu.sa; 6 Centre for Applied Physics and Radiation Technologies, School of Engineering and Technology, Sunway University, Bandar Sunway 47500, Selangor, Malaysia; d.a.bradley@surrey.ac.uk; 7 Department of Physics, University of Surrey, Guildford GU2 7XH, UK; 8 Radiation and Nuclear Safety Institute, Sudan Atomic Energy Commission, Khartoum P.O. Box 3001, Sudan

**Keywords:** radiation dosimetry, radiotherapy, medical LINAC, ionisation chamber, absorbed dose standards

## Abstract

This paper presents guidelines for the calibration of radiation beams that were issued by the International Atomic Energy Agency (IAEA TRS 398), the American Association of Physicists in Medicine (AAPM TG 51) and the German task group (DIN 6800-2). These protocols are based on the use of an ionization chamber calibrated in terms of absorbed dose to water in a standard laboratory’s reference quality beam, where the previous protocols were based on air kerma standards. This study aims to determine uncertainties in dosimetry for electron beam radiotherapy using internationally established high-energy radiotherapy beam calibration standards. Methods: D_w_ was determined in 6-, 12- and 18 MeV electron energies under reference conditions using three cylindrical and two plane-parallel ion chambers in concert with the IAEA TRS 398, AAPM TG 51 and DIN 6800-2 absorbed dose protocols. From mean measured D_w_ values, the ratio TRS 398/TG 51 was found to vary between 0.988 and 1.004, while for the counterpart TRS 398/DIN 6800-2 and TG 51/DIN 6800-2, the variation ranges were 0.991–1.003 and 0.997–1.005, respectively. For the cylindrical chambers, the relative combined uncertainty (*k* = 1) in absorbed dose measurements was 1.44%, while for the plane-parallel chambers, it ranged from 1.53 to 1.88%. Conclusions: A high degree of consistency was demonstrated among the three protocols. It is suggested that in the use of the presently determined dose conversion factors across the three protocols, dose intercomparisons can be facilitated between radiotherapy centres.

## 1. Introduction

Current dosimetry protocols for calibration of clinical high-energy photon beams are based on the standard adoption of absorbed dose to water (D_w_) [1,2,3,4]. Absorbed dose standards allow the use of a more straightforward formalism, providing fewer uncertainties compared to the previous air kerma protocols and a more robust system of primary standards for radiation measurements [5,6]. In dosimetry, there is a regular need to reflect upon progress made in seeking improved accuracy, high-precision radiotherapy dosimetry, an important part of which is the comparison of the different protocols that are widely adopted. Overall, the desire is to reduce discrepancies in measurements and to assist in dose standardization.

Following publication of these protocols some 20 years ago, a number of studies have sought to determine differences that might derive from their adoption and to provide insights into the origin of any such differences. Several studies have been conducted to compare dosimetry protocols and address the issue reducing uncertainties in external beam therapy [7,8,9].

In this study, we compared the use of three widely adopted internationally authoritative protocols, namely IAEA 398, DIN 6800-2 and the American Association of Physicists in Medicine (AAPM TG 51). We add to such efforts, making measurements through use of five ionization chamber types, all acknowledged to be suitable for electron dosimetry. These comprise three cylindrical and two plane-parallel chambers, and there is the additional possibility of clinical significance from chamber-to-chamber variations in dose determination.

TG 51 is a popular protocol in North America, DIN 6800-2 enjoys a greater presence in Europe and IAEA TRS 398 enjoys more global coverage; preference for a given code is almost certainly influenced by cultural factors, prior training, habit and familiarity. With such ad hoc choice of protocol, there is a need to provide dose conversion coefficients, sufficient to facilitate intercomparison of dosimetric measurements among radiotherapy centres. 

Several studies have compared the current absorbed dose standard to its predecessors, which were based on air kerma standards. Among current absorbed dose standards, studies have examined the effect of theoretical formulations on measurement results of absorbed dose to water. However, studies comparing the uncertainties arising from the measurement of absorbed dose using the current protocols are lacking.

### 1.1. Basic Dosimetry Formalism

According to IAEA TRS 398, the absorbed dose to water,$\;D_{w,Q}$, in a hospital beam quality (*Q*) is determined using an ion chamber calibrated in terms of absorbed dose to water at a cobalt-60 reference beam quality ($Q_{o}$) [1]:

(1) $$D_{w,Q}=M_{Q}N_{D,w,Q_{o}}k_{Q,Q_{o}}$$

where $k_{Q,Q_{o}}$ is the beam quality correction factor,$\;N_{D,w,Q_{o}}$ is the absorbed dose to water calibration factor and $M_{Q}$ is the corrected ionization chamber reading at the hospital. The beam quality correction factor $k_{Q}=k_{Q}^{\prime }k_{Q}^{\prime \prime }$, where $k_{Q}^{\prime }$ and $k_{Q}^{\prime \prime }$ are factors related to the beam quality and the ion chamber, respectively. The uncorrected dosimeter reading $M_{raw}$ is corrected to the effluence quantities according to:

(2) $$M_{Q}=M_{raw}k_{TP}K_{elec}k_{pol}k_{s}$$

where $k_{TP}$ is the correction factor for the ambient pressure temperature, $K_{elec}$ the calibration factor electrometer, $k_{pol}$ correction factor to account for change in the ion chamber polarity and $k_{s}$ correction factor to account for the ion recombination.

In the AAPM protocol TG 51, the absorbed dose to water ($D_{w}^{Q}$) at the hospital beam quality (Q) is determined using an ion chamber absorbed dose to water calibration factor ($D_{D,w}^{60\;Co}$) at a cobalt-60 reference beam quality ($Q_{o}$) [2]:

(3) $$D_{w}^{Q}=Mk_{Q,Q_{o}}D_{D,w}^{60\;Co}$$

where *M* is the corrected ion chamber reading, and $k_{Q,Q_{o}}$ is the beam quality correction factor. $k_{Q}=P_{gr}^{Q}\cdot k_{R_{50}}^{\prime }\cdot k_{ecal}$, where $k_{R_{50}}^{\prime }$ is the factor to convert the calibration factor to the actual radiation quality *R*_50_. $P_{gr}^{Q}$ accounts for ionisation gradient in the ionisation chamber, and $k_{ecal}$ is related to the radiation quality. 

In DIN 6800-2, the absorbed dose to water at the hospital beam quality (*Q*) is determined using an un-ion chamber calibrated in terms of N the absorbed dose to water calibration factor at a cobalt-60 reference beam quality ($Q_{o}$) [3]:

(4) $$D_{w}\left(P_{\mathrm{eff}}\right)=\mathrm{KNM}$$

M is the corrected reading of the ion chamber; K is the beam quality correction. ($K=k_{E^{\prime }}\cdot \;k_{E^{\prime \prime }}$), where $k_{E^{\prime }}$ and $k_{E^{\prime \prime }}$ are factors related beam quality and the ion chamber, respectively. Table 1. Presents summary of different annotations used in the three dosimetry protocols.

### 1.2. Other Correction Factors of the Ion Chamber Readings

Ionisation chamber readings are affected by the influence quantities that differ from the condition at the reference laboratory where it was calibrated. Correction factors are needed to correct for the nonreference conditions at the hospital environment. Measurements were made according to the method described in each protocol. 

For air density correction, the correction factor k is applied:

(5) $$k_{p}=\frac{\left(273.15+T\right)P_{o}}{\left(273.15+T_{o}\right)P}$$

where *P* and *T* are ambient pressure, and temperature and $P_{o}$ and $T_{o}$ are the reference pressure and temperature (in this study, 101.13 kPa and 20 °C).

The ion recombination correction factor accounts for incomplete collection of charges in the ionization chamber. In the TRS 398 and TG 51 dosimetry protocols, ion recombination correction factor is derived using the two-voltage technique. $k_{s}$ is set at the normal operating voltage V1 [1,2,3].

(6) $$k_{s}=a_{o}+a_{1}\left(\frac{M_{1}}{M_{2}}\right)+a_{2}{\left(\frac{M_{1}}{M_{2}}\right)}^{2}$$

where *M*_1_ and *M*_2_ are charges collected using polarizing voltages V1 and V1, respectively; V1/V2 > 3; and aj are coefficients used to determine Ps. In DIN, the ion recombination correction factor is determined using the equation introduced by [3]:

(7) $$k_{s}=1+0.54\frac{D_{i}d^{2}}{U}\;\mathrm{for}\;d=2.5\;\mathrm{mm}$$

where *D_i_* is the dose per pulse (mGy), *d* is the cavity height (mm) and *U* is the voltage.

The effect on a chamber reading because of using opposite polarity is corrected using ion chamber polarity correction factor $k_{pol}$ given by [1,2,3].

(8) $$k_{pol}=\left(\frac{\left|M_{+}\right|+\left|M_{\pm }\right|}{2M}\right)/{\left(\frac{\left|M_{+}\right|+\left|M_{\pm }\right|}{2M}\right)}_{Co_{60}}.$$

where *M* is the ion chamber reading obtained with the polarity used regularly, $M_{+}$ and $M_{-}$ are the ion chamber readings at positive and negative polarities.

### 1.3. Perturbation Factors

The sensitivity of an ionization chamber expressed in the form of an ionization chamber calibration factor depends on the water-to-air stopping power ratio of and the overall perturbation correction factor, P [1,2,3,4].

The perturbation factor corrects any departures from the ideal Bragg–Gray condition that may occur when a nonwater-equivalent wall is placed in water. P comprises the wall correction factor, P_wall_; fluence correction factor, P_fl_; central electrode correction factor, Pcel; and gradient correction factor, Pgr. For parallel plate chambers, wall (P_wall_) and the fluence perturbation (P_cav_) correction factors are assumed to be unity in all dosimetry protocols.

## 2. Materials and Methods

The experimental measurements were performed at the Department of Medical Physics, Institut Curie, Paris, France. Measurements were performed for three clinical electron beams delivered by two Varian medical linear accelerator types, LINAC 2300 C and LINAC 2100 C, having energies of 6, 12 and 18 MeV. The dose-dependent characteristics of the electron beams under study are presented in presented in Table 1. The repetition rate of the pulsed beams was 50 Hz, giving a dose rate of 200 MU/min for all beams, which was incident horizontally on a water phantom for measurements at reference condition specified in the relevant dosimetry protocol. A variable, stabilized high voltage supply, provided the polarizing voltage. The gantry and collimator were set at zero degrees.

### 2.1. Dosimetry Equipment

Dosimetry systems that include an ion chamber with an electrometer were manufactured and calibrated at the IBA dosimetry laboratory (IBA Dosimetry GmbH, Schwarzenbruck, Germany), with calibration traceable to the National Metrology Institute of the Federal Republic of Germany, PTB. Experimental measurements were conducted using IAEA TRS 398, AAPM TG 51 and DIN 6800-2 dosimetry protocols, and a set of five ionization chambers consisted of FC65-G type cylindrical chamber (S/N. 1630), two cylindrical farmer type CC13 and IC15 ion chambers (S/N. 8307 and 3560) and two parallel-plate ion chambers type NACP-02. The characteristics of the ionization chambers calibration coefficients are presented in Table 2.

For dose measurements, the following electrometers were deployed: Victoreen Model 530 electrometer and Keithley Model 350 electrometer (Elimpex-Medizintechnik, Spechtgasse 32, A-2340 Modelling, Austria). All measurements were made using Wellhofer Computerized water phantom (IBA Dosimetry GmbH, Schwarzenbruck, Germany).

### 2.2. Experimental Measurements

In dosimetry measurements, the radiation beam quality index (Q) is an important parameter used to determine the energy conversion factor; $k_{Q}$ required us to calculate the absorbed dose to water, as shown in Equations (1)–(3).

Plane-parallel chambers are the recommended ion chambers for absorbed dose measurement in radiotherapy using electron beams. NACP-02 plane-parallel chambers were cross-calibrated against the FC65-G type cylindrical reference chamber at each hospital beam energy before experimental measurements, as recommended [1,2].

The reference point for the plane-parallel chamber is on the inner surface of the entrance window at its centre, and for the cylindrical chamber, it is on the chamber axis at the centre of the chamber cavity volume [1,2,3]. Cylindrical chambers with Cobal-60 calibration factors were used for absorbed dose measurement in radiotherapy electron beam energies < 10 MeV [1,2,3,4].

D_w_ is determined at a reference depth of measurements (*Z_ref_*), the value of which depends on the 50% range of absorbed dose (*R*_50_). In TG 50 and TRS 398, the position of the reference point of the chamber for plane-parallel chambers is at the reference depth (*Z_ref_*); for the cylindrical chambers, it is at the effective point of measurements, 0.5 rcyl deeper than *Z_ref_*. The 50% range of ionization, *I*_50_, is measured and transformed into *R*_50_ using the following equation:

(9) $$R_{50}=1.029I_{50}-0.06$$

Reference depth of measurement (*Zref*) is calculated as follows:

(10) $$Z_{ref}=0.6\cdot R_{50}-0.1\;(\mathrm{cm})$$

Typical values of *R*_50_, *Z_ref_*, and *Z_max_* used for measurements are presented in Table 3.

The experimental setup for all-electron beams was 10 × 10 cm^2^ and 100 cm SSD. The depths were set at *Z_ref_* and *D_max_* for all energies depending on each protocol. The gantry and collimator were set at zero degrees. Using TG 51, measurements were performed at the same reference depth of 10 cm. For TRS 398 and DIN 6800-2, measurements were performed at the two reference depths of 5 cm (for 6 MeV) and 10 cm (for 20 MeV). All measured doses were transformed into the doses at maximum depth (*Z_max_*) using the central axis percentage depth dose (*PDD*) data, according to Equation (11).

(11) $$D_{w,Q}\left(Z_{max}\right)=100\cdot D_{w,Q}\left(Z_{ref}\right)/PDD\left(Z_{ref}\right)$$

### 2.3. Measurement Uncertainty

Uncertainties in measurements results are determined as standard deviations evaluated by either Type A methods based on statistical observations or Type B methods based on means other than statistical methods. The combined uncertainty of the results of the two evaluation methods is determined using error propagation. Thus, the combined uncertainty in the absorbed quantity to water calculated according to IAEA TRS 398 (Equation (1) can be expressed as follows [10,11]:

(12) $$\frac{u\left(D_{w,Q}\right)}{D_{w,Q}}=\sqrt{{\left(\frac{u\left(M_{Q}\right)}{M_{Q}}\right)}^{2}+{\left(\frac{u\left(N_{D,w,Q_{o}}\right)}{N_{D,w,Q_{o}}}\right)}^{2}+{\left(\frac{u\left(k_{Q,Q_{o}}\right)}{k_{Q,Q_{o}}}\right)}^{2}}$$

where $\frac{u\left(M_{Q}\right)}{M_{Q}}$,$\frac{u\left(N_{D,w,Q_{o}}\right)}{N_{D,w,Q_{o}}}$,$\left(\frac{u\left(k_{Q,Q_{o}}\right)}{k_{Q,Q_{o}}}\right)$ are the relative standard uncertainties in the corrected hospital measurement, absorbed dose to water calibration factor and the beam quality correction factors, respectively. The relative uncertainty ($\frac{u\left(M_{Q}\right)}{M_{Q}}$) can be written as:

(13) $$\frac{u\left(M_{Q}\right)}{M_{Q}}=\sqrt{{\left(\frac{u\left(M_{raw}\right)}{M_{raw}}\right)}^{2}+{\left(\frac{u\left(k_{TP}\right)}{k_{TP}}\right)}^{2}+{\left(\frac{u\left(K_{elec}\right)}{K_{elec}}\right)}^{2}+{\left(\frac{u\left(k_{pol}\right)}{k_{pol}}\right)}^{2}+{\left(\frac{u\left(k_{s}\right)}{k_{s}}\right)}^{2}}$$

where $\frac{u\left(M_{raw}\right)}{M_{raw}}$, $\frac{u\left(k_{TP}\right)}{k_{TP}}$, $\frac{u\left(K_{elec}\right)}{K_{elec}}$,$\;\frac{u\left(k_{pol}\right)}{k_{pol}}$,$\;{\left(\frac{u\left(k_{s}\right)}{k_{s}}\right)}^{2}$ are the relative standard uncertainties for uncorrected ion chamber reading (*M_w_*), temperature and pressure correction factor (*k_TP_*), electrometer calibration factor (*k_elec_*), polarity correction factor (*k_pol_*) and ion recombination correction factor (*k_s_*), respectively. The measurement results’ overall uncertainties were quoted as expanded uncertainty at 68% confidence level with coverage factor (*k* = 1) [12].

## 3. Results

Results are presented for the D_w_ measured using three cylindrical and two plane-parallel ion chambers in concert with absorbed dose-based protocols. To compare the three protocols, measurements were made in reference conditions given in each protocol.

Table 4 presents the beam quality correction factors and data used to determine D_w_. Table 5 presents the polarity effect (*k_pol_*), ion recombination (*k_s_*) and temperature and pressure (*k_TP_*) correction factors. Correction factors for the same type of chamber (IC15/CC13) were made using a single chamber and then applying them to the other, since these chambers have the same perturbation factors [1]. For NACP chambers, the contribution of the chamber factor in *k_Q_* is significant, so both chambers were used for measurement.

In Table 6, the absorbed dose to water ratios is given between TRS 398, TG 51 and DIN 6800-2. From mean measured D_w_, the ratio TRS 398/TG 51 was found to vary between 0.988 and 1.004, while for the counterpart TRS 398/DIN 6800-2 and TG 51/DIN 6800-2, the variation ranges were 0.991 to 1.003 and 0.997 to 1.005, respectively.

**Figure 1:**
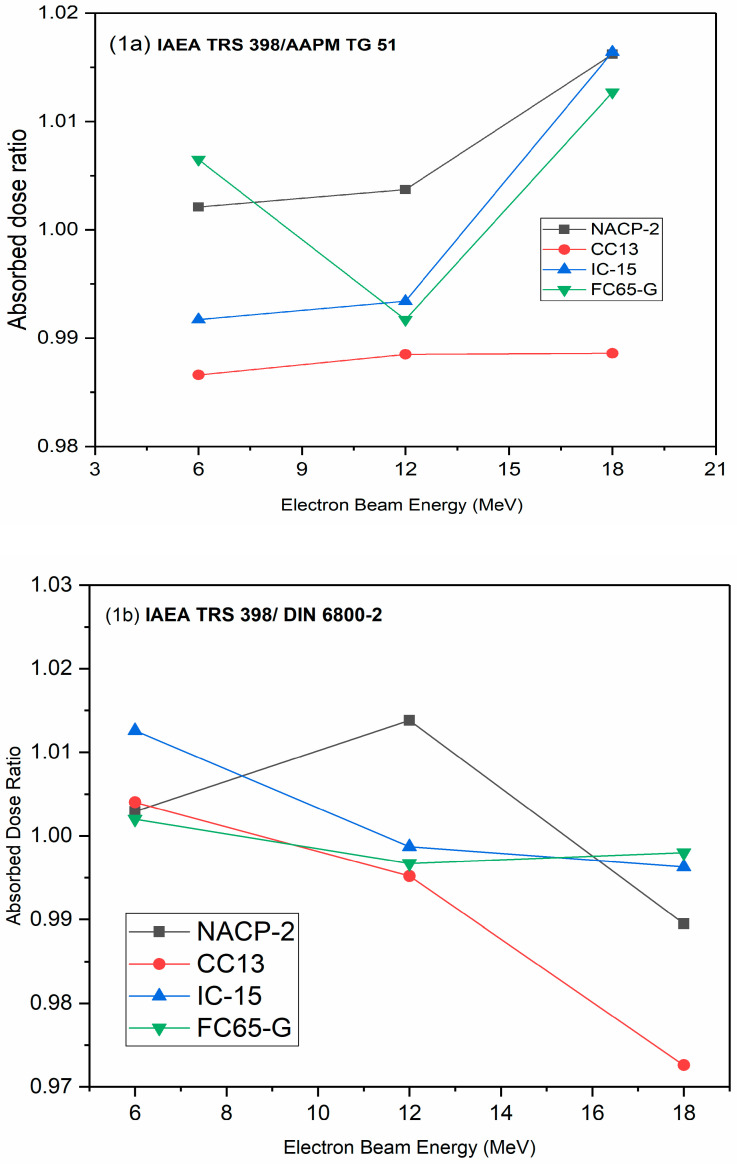

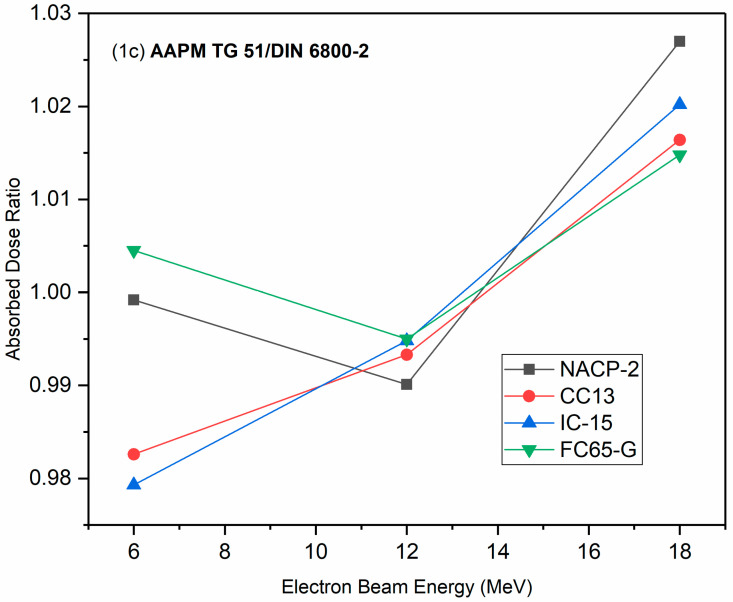
Ratio of the absorbed dose to water values obtained using five ionization chambers in three electron beams: (**a**) IAEA TRS 398/AAPM TG 51; (**b**) IAEA TRS 398/DIN 6800-2; (**c**) AAPM TG 51/DIN 6800-2.

In Figure 1a–c, ratios are presented for the D_w_ obtained using five ion chambers in the studied electron beams. The absorbed dose measured using NACP chamber differed by about 1.5% in TRS 398 and TG 51 versus that of DIN 6800-2, while the corresponding figure obtained using the FC65-G chamber deviated by about 1.6%. These values agree with the results previously reported in the literature [13,14,15].

Our results show a high degree of consistency with the measurement uncertainty between the IAEA TRS 398 and DIN 6800-2. The most significant uncertainties are presented for plane-parallel chambers (NACP-02). Zink and Wulff [16] reported that this is mainly ascribed to the significant uncertainties of the mean ionization energy for the graphite used in the ionization chamber design and the thickness of the chamber entrance wall. Currently obtained uncertainties are in the range 1.44–1.88%, which is in line with uncertainties reported in the most recent IAEA publication (1.4–2.1%) [8].

Castro et al. [17] studied the uncertainty in absorbed dose to water in calibration of high-energy radiotherapy. These authors reported uncertainty (k = 1) in the absorbed dose to water of 1.5% for an electron beam. In another study, de Prez et al. [18] reported a combined standard uncertainty (k = 1) for the absorbed dose to water in electron beams in the range 1.6–1.8%. These values compare well with the results of the current study.

## 4. Discussion

Absorbed dose measurements using IAEA TRS 398, AAPM TG 51 and DIN 6800-2 protocols agree within 2% for the electron beam energies under study. Different factors cause discrepancies in the measured absorbed dose values.

The results of parallel plate chambers are expected to differ from those of cylindrical chambers due to differences in the perturbation correction factors included in the radiation quality correction factors [15]. Because of this, correction factors for beam quality vary depending on the type of ionization chamber used. Another difference stems from the use of different methods to evolve influence quantities that correct for nonreference conditions. The results showed good agreement for FC65-G and the NACP-02 chambers. The discrepancy in the measurement results was up to 1.3% for the FC65-G ionization chamber. Comparing NACP chambers with TRS 398 and TG 51 to DIN 6800-2, the discrepancy in measurement results was up to 1.5%, while the corresponding figure for FC65-G chambers was about 1.6% (Figure 1).

Recently, several studies were also performed to compare protocols based on absorbed dose standards. The common goal was to improve dosimetric accuracy and to report discrepancies. In conformity to our results, the agreement between the two protocols was more pronounced for NACP-02 plane-parallel chambers. A similar agreement was shown by Zakaria and Schutte, who reported a deviation up to 1.6% for the IAEA TRS 398, AAPM TG 51 and DIN 6800-2 [14].

Table 7 gives the estimated % uncertainty (relative) in measurements of D_w_, the use of high-energy electron beam clinical accelerators, and the current absorbed dose-based protocols. Concerning the required accuracy in radiotherapy, the aim is to limit the increase in toxicity to 3%. Dose uncertainties (σD) would need to be kept to <5% [12]. The current uncertainties fall with the stated limits.

## 5. Conclusions

The study found that the absorbed dose to water conversion coefficients of the three protocols were quite similar, indicating that the measurement results were very consistent. The results obtained are expected to facilitate intercomparison of measured results between hospitals using different protocols. The main sources of uncertainty in the results were chamber type and electron energy, which were attributed to differences in the perturbation factors used in the various protocols. The results reveal that a significant fraction of the uncertainties come from beam quality correction factors. These uncertainties could be reduced by having standards provide calibration of ion chambers in user beams. Results are essential for facilitating intercomparison between radiotherapy centres that use different dosimetry protocols.

## Figures and Tables

**Table 1:** Summary of different annotations used in the three dosimetry protocols.

Factor	Annotations		
	IAEA TRS 398	AAPM TG 51	DIN 6800-2
Reference radiation beam quality (cobalt-60)	$Q_{o}$	60*Co*	-
Hospital radiation beam quality	*Q*	*Q*	-
absorbed dose to water	$D_{w,Q}$	$D_{w}^{Q}$	$D_{w}\left(P_{\mathrm{eff}}\right)$
absorbed dose to water calibration factor	$N_{D,w,Q_{o}}$	$D_{D,w}^{60\;Co}$	N
beam quality correction factor	*k_Q_*	*k_Q_*	K
the corrected ion chamber reading	$M_{Q}$	M	M

**Table 2:** Physical characteristics of plane-parallel and cylindrical ionization chambers.

Chamber	Type	Entrance Window		Cavity Wall		Cavity Volume (cc)	Waterproof (Y/N)	Preference
		Material	*d* (mm)	Material	Thickness (mm)			
NACP-02 (A)	parallel plate	0.17 mm mylar	7	NA	NA	NA	no	Absolute dosimetry
NACP-02 (B)	parallel plate	0.17 mm mylar	7	NA	NA	NA	no	Absolute dosimetry
IC15	cylindrical	NA	NA	C552	0.4	0.13	yes	Relative dosimetry
CC13	cylindrical	NA	NA	C552	0.4	0.13	yes	Relative dosimetry
FC65-G	cylindrical	NA	NA	Graphite	0.4	0.65	yes	Absolute dosimetry

**Table 3:** Characteristics of the electron beams used.

Beam Energy (MeV)	*R*_50_ (cm)	*Z_ref_* (cm)	*Z_max_* (cm)	
			TG 51, DIN 6800-2	TRS 398
6	2.3	1.3	1.3	1.4
12	4.9	2.9	2.8	3.0
18	7.6	2.5	4.5	4.6

**Table 4:** Beam quality correction factors used to calculate absorbed dose to water using the TRS 398, TG 51 and DIN 6800-2 absorbed dose-based protocols.

Chamber	Beam Energy (MeV)	$N_{D,w}^{60Co}$	$k_{Q}$		
			TRS 398	TG -51	DIN 6800-2
NACP-02 (13505)	6	1.748 × 10^8^	0.9268	0.9302	0.9293
	12		0.8990	0.9059	0.9023
	18		0.8824	0.8881	0.8845
NACP-02 (13703)	6	1.58 × 10^8^	0.9268	0.9302	0.9293
	12		0.8990	0.9059	0.9023
	18		0.8824	0.8881	0.8845
IC15	6	1.3633 × 10^8^	0.9350	0.9203	0.9395
	12		0.9172	0.9093	0.9138
	18		0.9082	0.9005	0.9060
FC65-G	6	4.738 × 10^7^	0.9350	0.9218	0.9376
	12		0.9164	0.9092	0.9128
	18		0.9082	0.8984	0.9051

**Table 5:** Polarity effect (*k_pol_*), ion recombination (*k_s_*) and temperature and pressure correction factors (*k_TP_*).

Correction Factor	6 MeV			12 MeV			18 MeV		
	TRS	TG 51	DIN 6800-2	TRS	TG 51	6800-2	TRS	TG 51	6800-2
NACP									
*k_pol_*	0.9994	0.9994	0.9996	0.9993	0.9993	0.9990	0.9981	0.9981	0.9989
*k_s_*	1.0096	1.0099	1.0089	1.0107	1.0110	1.0028	1.0094	1.0096	1.0113
*k_TP_*	1.0169	1.0100	1.0169	1.0169	1.0100	1.0169	1.0169	1.0100	1.0169
CC13									
*k_pol_*	1.0025	0.9922	1.0027	1.0009	1.0008	1.0010	1.0010	1.0005	1.0010
*k_s_*	1.0244	1.0365	1.0252	1.0246	1.0247	1.0255	1.0256	1.0252	1.0259
*k_TP_*	1.0168	1.0099	1.0168	1.0168	1.0099	1.0168	1.0169	1.0099	1.0168
FC65-G									
*k_pol_*	1.0014	1.0013	1.0011	1.0001	1.0001	0.9999	1.0001	0.9988	0.9992
*k_s_*	1.0175	1.0181	1.0182	1.0177	1.0181	1.0180	1.0184	1.0188	1.0197
*k_TP_*	1.0291	1.0221	1.0291	1.0291	1.0221	1.0291	1.0291	1.0221	1.0291

**Table 6:** Conversion factors for absorbed dose to water between the TRS 398, TG 51 and DIN 6800-2 dosimetry protocols using NACP-02, CC13, IC15 and FC65-G ionisation chambers.

Ionisation Chamber	Beam Energy	IAEA TRS 398/ AAPM TG 51	IAEA TRS 398/ DIN 6800-2	AAPM TG 51/ DIN 6800-2
NACP-02	6	1.0021	1.0029	0.9992
	12	1.0037	1.0138	0.9901
	18	1.0162	0.9895	1.0270
CC13	6	0.9866	1.0040	0.9826
	12	0.9885	0.9952	0.9933
	18	0.9886	0.9726	1.0164
IC15	6	0.9917	1.0126	0.9793
	12	0.9934	0.9987	0.9948
	18	1.0164	0.9963	1.0202
FC65-G	6	1.0065	1.0020	1.0045
	12	0.9917	0.9967	0.9950
	18	1.0127	0.9980	1.0148

**Table 7:** Relative uncertainty (%) associated with measurements of the absorbed dose to water, D_w_, in energy electron beam radiotherapy using the IAEA TRS 398 and DIN 6800-2 dosimetry protocols.

Influence Quantities		Evaluation	Cylindrical Chamber		Plane-Parallel Chamber	
			TRS 398	DIN 6800-2	TRS 398	DIN 6800-2
ND, W	Chamber certificate	B	0.55	0.55	0.55	0.55
Measurement depth	Calculated	B	0.46	0.46	0.46	0.46
*k_p_*	Calculated	A/B	0.04	0.04	0.12	0.12
*k_TP_*	Calculated	A/B	0.01	0.01	0.01	0.01
*k_s_*	Calculated	B	0.04	0.04	0.12	0.12
*k_Q_*	IAEA TRS 398	B	1.2	-	1.7	-
*k_E_*	DIN 6800-2	B	-	1.2	-	1.3
Meter stability	Dosimeter Manual	B	0.28	0.28	0.28	0.28
Meter reading	Calculated	A	0.2	0.2	0.2	0.2
Combined standard uncertainty (*k* = 1)			1.44	1.44	1.88	1.53

## Data Availability

Data available on request.
